# Comprehensive Management of Rheumatic Diseases Affecting the Temporomandibular Joint

**DOI:** 10.3390/diagnostics11030409

**Published:** 2021-02-27

**Authors:** Lauren Covert, Heather Van Mater, Benjamin L. Hechler

**Affiliations:** 1 Department of Pediatrics, Division of Rheumatology, Duke University Hospitals, Durham, NC 27710, USA; lauren.tam@duke.edu (L.C.); heather.vanmater@duke.edu (H.V.M.); 2 Department of Surgery, Division of Plastic, Maxillofacial, and Oral Surgery, Duke University Hospitals, Durham, NC 27710, USA; 3 Department of Head and Neck Surgery and Communication Sciences, Duke University Hospitals, Durham, NC 27710, USA

**Keywords:** temporomandibular joint, temporomandibular disorder, rheumatic disease, juvenile idiopathic arthritis, rheumatoid arthritis, inflammatory arthritis

## Abstract

The temporomandibular joint (TMJ) is a synovial joint and thus is vulnerable to the afflictions that may affect other joints in the fields of rheumatology and orthopedics. Too often temporomandibular complaints are seen strictly as dental or orofacial concerns. Similarly, patients with known rheumatic disease may not have their TMJs included in routine screening and monitoring protocols. The purpose of this review is to highlight the rheumatic conditions likely to affect the TMJ and outline medical and surgical management in these patients with a focus on the need for continued patient reassessment and monitoring.

## 1. Introduction

The temporomandibular joint (TMJ) is a synovial joint of high functional significance. Although TMJ disorders (TMD) are often thought of as dental or orofacial phenomena, we must not forget that true intracapsular, diacapitular TMJ disease is an arthropathy. When significant intracapsular TMJ damage and dysfunction are present, occlusal imbalance or myofascial pain are never the cause, and an underlying arthropathy must be investigated. Similarly, patients with known rheumatic diseases should be investigated for TMJ involvement. Furthermore, it should be kept in mind that the young to middle-aged female population is the typical patient population presenting with autoimmune disorders and TMD [1]. It is thus crucial that rheumatologists, dentists, oral and maxillofacial surgeons (OMS), and head and neck surgeons are able to independently understand the TMJ’s place in manifesting rheumatic diseases.

One-fifth to one-fourth of Americans report a doctor-diagnosed arthritic condition [2], with the Centers for Disease Control and Prevention (CDC) confirming that approximately 25% of US adults suffer from arthritis [3]. The World Health Organization (WHO) has quantified the worldwide morbidity of some of the most common arthritic conditions, with 25% of those with osteoarthritis (OA) unable to perform major daily activities of living, and 50% of those with rheumatoid arthritis (RA) unable to perform full-time job activities within 10 years of disease onset [4]. Although certain autoimmune rheumatic diseases are less common, their individual patient morbidity can be significantly more serious. The terminology used to refer to these musculoskeletal conditions is often inconsistent and confusing. Joint diseases should collectively be referred to as “arthropathies”, although in the English language the term “arthritis” has been used extensively and in disparate contexts. Throughout this manuscript, “rheumatic diseases” is used to refer primarily to inflammatory autoimmune conditions, unless otherwise indicated (e.g., osteoarthritis).

Comprehensive management of the TMJ in rheumatic diseases is based upon the initial understanding of four important principles:

### 1.1. All TMD Should Be Considered as Potentially Secondary to an Underlying Systemic Condition

The first principle in understanding how to manage TMD in rheumatic diseases is simply the realization that TMD may be a manifestation of an underlying rheumatic condition. It can again not be overemphasized that true intracapsular, diacapitular TMJ disease is an arthropathy. Similar to how visualizing an oral lesion concerning for squamous cell carcinoma should prompt questions of weight loss or dysphagia, TMJ signs or symptoms should prompt questions of other joint involvement, constitutional symptoms, or synchronously or metachronously identified organ involvement.

### 1.2. TMD Presents Differently in Rheumatic Diseases Than in Non-Rheumatic TMD

Clinicians who frequently treat TMD in non-rheumatic patients are accustomed to a pattern of linear disease progression consistent with the Wilkes classification. Indeed, Wilkes commented on the “strong relation to the time course of the organic lesions present”, with clinical, radiographic, and pathologic findings correlating well [5]. In rheumatic diseases—particularly autoimmune conditions requiring various medical interventions and those present in children and adolescents—the temporal progression of TMD may be unexpected, and a correlation between clinical and radiographic findings is not to be assumed.

### 1.3. Temporomandibular Joint Disease Is a Continuum

In science in general and medicine in particular, “cut offs” are often chosen by statistical optimization and therefore may not have strict clinical correlations. In reality, clinical continua are much more common. Accordingly, it is thus much more clinically relevant to follow parameters *within* an individual patient *across time* than to compare parameters *across* patients *at a single time point*. Patients who develop more severe systemic symptoms will be more likely to develop more severe TMJ symptoms.

### 1.4. Rheumatic Diseases Can also Manifest as Parotid Abnormalities

**Figure 1:**
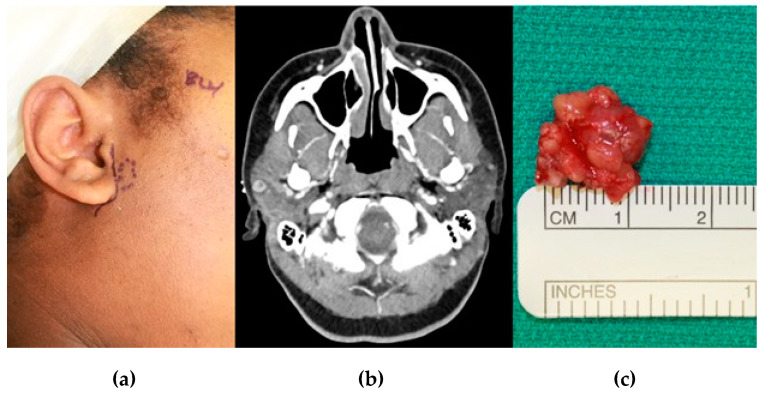
A 26-year-old female presented for evaluation with chief complaints of right TMJ popping and pre-auricular pain for one year. She reported a strong family history of both systemic lupus erythematosus and Sjogren Syndrome. C-reactive protein was elevated and anti-nuclear antibodies showed speckled and homogenous patterns in high titers. (**a**) Dotted line indicates location of pain per patient; (**b**) CT was intentionally obtained with contrast to evaluate for TMJ and soft tissue abnormalities. Note an intra-parotid lesion lying immediately lateral to the mandibular condyle; (**c**) Intra-parotid lesion removed and found to be a basal cell adenoma. The patient’s symptoms resolved after treatment.

Although not the topic of this review, it should be realized that rheumatic conditions frequently affect not only the TMJ but the anatomically proximate parotid glands. Indeed, those with rheumatoid arthritis (RA) have been shown to have abnormal intra-parotid lymph nodes as compared to controls [6]. This gives further credence to principle one, that pre-auricular signs and symptoms should always be investigated in the context of known or potential rheumatic conditions. A corollary to this line of thought is that should a CT of the face be planned for evaluation of possible TMJ pathology, addition of IV contrast should be considered—assuming the patient’s medical condition allows—in the event that TMJ, infratemporal fossa, or parotid soft tissue pathology is the true etiology (Figure 1).

## 2. Rheumatic DISEASES Affecting the Temporomandibular Joint

**Figure 2:**
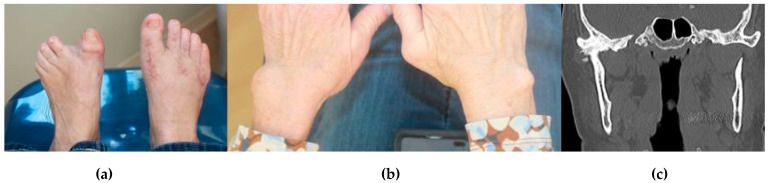
A 56-year-old female presented for evaluation with chief complaints of right TMJ pain and limited mandibular opening. Her history was most notable for long-standing RA refractory to multiple medications. (**a**) Bilateral toe involvement requiring Hoffman procedure; (**b**) Bilateral wrist involvement; (**c**) CT of the face showed early unilateral right TMJ ankylosis, lateral pannus formation, and heterotopic bone formation. The left TMJ was completely normal.

As will be seen, many rheumatic diseases can affect the TMJ. Original reviews suggesting that those with rheumatoid arthritis (RA) will have bilateral, symmetric TMJ involvement while those with seronegative spondyloarthropathies (SNS) will have unilateral disease should be viewed with caution [7] (Figure 2). For example, a review of the currently reported TMJ ankylosis cases in ankylosing spondylitis (AS) patients in the English literature suggests that approximately half presented with bilateral ankylosis [8]. A corollary of this line of thought is the fact that no radiographic findings or clinical signs or symptoms are pathognomonic for a specific rheumatologic disease.

It should also be noted that the more infrequent a specific set of conditions is reported in the literature (e.g., significant TMJ disease in patients with a specific rheumatologic diagnosis), the more anecdotal the reports become. For example, a cursory review of the literature on TMJ disease in rheumatic diseases reveals many case reports of TMJ ankylosis; however, the astute reader must realize the entire reason such reports are worthy of case reports is their overall infrequency amongst a given population.

An abbreviated reference of diagnostic criteria for each condition which can manifest with TMJ dysfunction is presented in Table 1. 

### 2.1. Juvenile Idiopathic Arthritis

Juvenile idiopathic arthritis (JIA), formerly known as juvenile rheumatoid arthritis (JRA), is seen by many as the most concerning rheumatic condition associated with TMJ dysfunction given the risk of dentofacial deformity in the growing child. Consequently, it is the most studied rheumatic condition causing TMJ dysfunction. Its diagnosis per the International League of Associations for Rheumatology (ILAR) requires six weeks of arthritis in a patient under 16 years of age with the exclusion of other etiologic diagnoses [9]. Seven subcategories of the disease are recognized. It is reported that 17–87% of JIA patients will have TMJ involvement [10]. Of particular interest is that in JIA patients with acute TMJ arthritis up to 71% of cases may be asymptomatic and up to 63% may have normal findings on clinical exam [11]. Indeed, even when ultrasonic or MRI evaluation confirms joint effusion in these patients, the vast majority have been shown to be asymptomatic [12,13]. Because by definition the disease process begins in childhood or adolescence, the risk of dentofacial deformity is substantial.

### 2.2. Systemic Sclerosis/Scleroderma

Systemic sclerosis (SSc) is a heterogeneous group of disorders which like JIA can manifest in childhood (e.g., localized scleroderma) but is much more common in adults. For decades the mandible has been documented as a bone affected by the disease, both directly and indirectly [14,15]. Compared to the general population, even asymptomatic patients with SSc have decreased mandibular range of motion, although this can be confounded by soft tissue thickening resulting in micrognathia [16]. Although less common than other rheumatic diseases, SSc may be the rheumatic condition most associated with TMJ signs and symptoms, with multiple sources reporting >90% of SSc patients with TMJ signs and symptoms [17,18].

### 2.3. Rheumatoid Arthritis

Rheumatoid arthritis is characterized by polyarticular, erosive synovitis that is often relatively symmetrical and may present with significant extra-articular organ disease, a point made clear when it is recognized that those with RA have shorter lifespans than healthy controls [19]. It is reported that 5–86% of RA patients will have TMJ involvement, with 20–40% as a relatively consistent finding [20]. Similar to JIA, asymptomatic patients often have significant disease demonstrable on three-dimensional imaging. It has even been suggested that those with RA who are asymptomatic actually have a *higher* likelihood of TMJ degenerative disease detected on CT than symptomatic patients [21]. In contradistinction, symptoms may occur prior to overt TMJ signs, making disease monitoring via C-reactive protein (CRP), erythrocyte sedimentation rate (ESR), and number of involved systemic joints important [22,23]. The presence of anti-cyclic citrullinated protein antibodies has been shown to be significantly associated with development of TMD in RA patients [24]. Cervical spine involvement also appears to increase the likelihood of TMJ disease [25]. A consistent finding in RA patients with TMJ involvement is the predominant sign and symptom being TMJ sounds [17,26], with disease severity (defined by number of edematous joints) associated with TMJ sounds [19].

### 2.4. Systemic Lupus Erythematosus

Systemic lupus erythematosus (SLE) has for decades been seen as the quintessential autoimmune disorder. Compared to the other rheumatic diseases affecting the TMJ, patients with SLE are less likely to have TMJ signs or symptoms, and there is conflicting data as to whether their signs and symptoms are different from control populations [17,20,27]. A now classic study by Jonsonn compared 37 SLE patients to 37 dental patients (controls) and found significantly worse signs, symptoms, and radiographic condylar flattening in the SLE patients; however, the majority of the SLE cohort had long-standing disease, all but one had systemic arthritis and arthralgia, and radiographic TMJ changes were significantly more common in patients with renal involvement, suggesting that the high frequency of TMJ complaints may represent an overall more active SLE population. [28]. A more recent study did indeed correlate more severe TMJ dysfunction in SLE with increased number of immunosuppressive medications, presumably a surrogate for disease activity [29]. SLE is one of the few conditions where “avascular” or “aseptic” necrosis of the TMJs is mentioned [30,31,32,33]; however, most of these reports come from single groups without histologic analysis of condylar specimens with the only assumption being that because patients have been on glucocorticoids avascular necrosis is likely. Conversely, what is much more likely is inflammatory arthritic destruction.

### 2.5. Axial Spondyloarthritis (Ankylosing Spondylitis, Non-Radiographic Axial Spondyloarthritis)

Both ankylosing spondylitis (AS) and non-radiographic axial spondyloarthritis (nr-axSpA) are subcategories of the umbrella diagnosis of axial spondyloarthritis (axSpA). Both are considered SNS processes. As the name implies, axSpA primarily involves the axial skeleton, either with (AS) or without (nr-axSpA) plain radiographic evidence of disease. AS and nr-axSpA may be distinct disease phenotypes or simply the spectrum of a single underlying disease process, as over the course of five years 20% of nr-axSpA cases develop radiographic evidence of disease [34]. The majority of axSpA patients are HLA-B27 positive, although this test is not completely sensitive or specific for the disease [35]. The TMJ is reported to be involved in 3–22% of patients, with the literature mainly focusing on patients with AS [36]. A general pattern observed in the cases of TMJ ankyloses in axSpA patients is that (1) the rheumatologic diagnosis is often made many years prior to TMJ dysfunction and (2) essentially all patients developing TMJ ankylosis previously had developed cervical spine fusion [37].

### 2.6. Psoriatic Arthrits

Psoriatic arthritis (PsA) is also an SNS and is a disease process originally said to be found in 5–7% of patients with psoriasis [38] but now thought to occur in 15–25% given the increased awareness and diagnosis of the disease [39]. The clinical patterns of the arthritic component most specific to PsA, as originally described [40], include distal interphalangeal (DIP) arthritis and arthritis mutilans (destructive arthritis), although other patterns may be present with significant overlap to other conditions, most notably RA. Although the TMJ is an infrequently involved joint in PsA, it has indeed been described as the first joint involved in PsA [41]. Because of the relatively low number of reports of PsA affecting the TMJs, firm conclusions on prevalence are difficult to make [8], although a recent review has suggested approximately one-third of PsA patients have TMJ symptoms [42]. Review of reports to date, however, do suggest a tendency for those with PsA and subsequent TMD to have worse disease and a significant erosive component, possibly not surprising given the destructive arthritic pattern present in many with severe PsA [38,43].

### 2.7. Others

#### 2.7.1. Osteoarthritis

Unlike the disorders described thus far, osteoarthritis (OA) is not a primary autoimmune inflammatory condition but a disease process marked by mechanical breakdown in the setting of abnormal forces or abnormal response to normal forces, with or without the presence of inflammation. Abnormal forces can be of increased magnitude (microtrauma) or increased frequency (microtrauma) [44], or normal forces can be applied to impaired articular cartilage or an abnormal disc-condyle complex [45]. Consequently, unilateral TMJ OA is often associated with asymmetric anatomy, asymmetric masticatory forces, or previous unilateral injury [46]. Unlike the axial or appendicular skeleton, obesity and occupation are not necessarily associated with OA of the TMJ. The diagnosis of TMJ osteoarthritis should, however, mirror the American College of Rheumatology (ACR) classification criteria for OA of the knee and hip: pain should be a primary symptom; joint stiffness, limited mobility, and crepitus will likely be present; radiographic evidence of erosion, subchondral cysts, subchondral sclerosis, and osteophytes are common; and elimination of autoimmune or infectious causes should be ensured [47,48].

#### 2.7.2. Fibromyalgia

Although fibromyalgia (FM) is not a cause of intra-articular TMD, patients with FM often present with signs and symptoms concerning for inflammatory articular disease including pre-auricular pain, pain on mandibular function, limited mouth opening, and diurnal change in symptoms. At least one study has gone as far as to suggest that all patients with FM present with pain when the TMJs and retrodiskal tissues are palpated [49]. A recent systematic review revealed a strong association between FM and TMD; however, the overwhelming association was with regard to complaints of pain, particularly masticatory muscular pain [50]. In this way, the FM patient often has a higher symptom burden relative to any radiographic abnormality while the inflammatory arthritis patient is more likely to have a lower symptom burden relative to the degree of radiographic joint disease. It should be noted that TMJ arthritic disease can present in patients with FM, but FM is not the etiology.

#### 2.7.3. Idiopathic Condylar Resorption

Although not a rheumatic inflammatory disease, idiopathic condylar resorption (ICR) must be mentioned as it presents nearly exclusively in adolescent and young women and thus demographically overlaps the patient population represented by systemic rheumatic conditions. Indeed, the original discussions on this phenomenon highlighted similarity to autoimmune resorption [51], although further investigations also emphasized what is now generally accepted as the role of hormones such as estrogen, prolactin, and endogenous steroids in this process [52]. Although ICR is usually symmetric, unlike rheumatic diseases it is not autoimmune and usually not inflammatory in nature, evidenced by the typical lack of synovitis and joint effusion on MRI even in the setting of active condylysis [53]. One frequently propagated misconception is that ICR is usually asymptomatic [54], when surveys actually suggest that the majority of ICR patients present with TMJ pain and myofascial pain [55]. ICR thus becomes a diagnosis of exclusion when symmetric condylysis is appreciated in a female patient whose rheumatologic work-up is otherwise negative.

## 3. Systemic Management of Rheumatic Diseases

While there are different types of inflammatory arthritides, as described above, systemic management across these distinct conditions share a similar approach and classes of medication including non-steroidal anti-inflammatory drugs (NSAIDs), corticosteroids, conventional and biologic disease-modifying antirheumatic drugs (DMARDs). Empirical practice with systemic treatment has beneficial effects on TMJ arthritis [56]. Goals of therapy for TMJ arthritis are similar to the treatment of arthritis in general—the cessation and prevention of joint damage, suppression of systemic disease, and eventual remission off medications [57]. Treatments for inflammatory arthritis are individualized based on severity of disease, number of joints involved, physical limitations and potential for joint damage [53]. 

Most of the literature regarding treatment of inflammatory TMJ arthritis with systemic medication is specific to JIA with a focus on an approach to normalize mandibular growth, reduce MRI-verified inflammation, and preserve osseous TMJ morphology. Current biologic medications have significantly decreased the extent of disability and need for major surgeries and joint replacement in JIA [57]. A retrospective study of 38 patients with JIA involving the TMJ, who were receiving systemic therapy, showed less severe osseous deformity and maintained normal mandibular ramus growth at 2 year follow up compared to baseline MRI. This contrasted to cohort studies with corticosteroid TMJ injections, in which TMJ deformity deteriorated and mandibular ramus growth was impaired [58]. 

Generally, however, there has been little evidence to guide management for TMJ arthritis. Most randomized controlled trials of DMARDs have not included TMJ involvement as an outcome, and there is minimal prospective data on medical therapy [59]. Consensus on treatment is lacking. In 2014, an 87-center multinational survey of pediatric rheumatologists worldwide showed that first-line treatment of TMJ arthritis varied with NSAIDs in 33%, non-biologic DMARDs in 36%, anti-TNF medication in 5%, and intra-articular steroid injection in 26% [60]. Furthermore, a cross-sectional survey of 52 academic OMS in the US revealed that the majority (81%) of JIA patients were being treated on average with 1–2 systemic medications, 13% on 3–4 medications and only 5% on no systemic medications [61]. It is worth noting that even with optimal medical management for peripheral arthritis in JIA, the TMJ is the most common joint that does not respond to initial therapy. Retrospective studies suggest that response to medical therapy of the TMJ may lag behind that of other joints for unclear reasons [62]. Consensus on treatment of TMJ arthritis in JIA is currently in development amongst pediatric rheumatologists within the US based on expert opinion. 

### 3.1. NSAIDs

NSAIDs such as naproxen, ibuprofen, and indomethacin are commonly used as an initial therapy in inflammatory arthritis with or without TMJ involvement. NSAIDs inhibit cyclooxygenase (COX)-2 activity, reducing cytokine-induced destruction of the extracellular matrix of the TMJ [63]. While often part of a maintenance medication regimen, NSAIDs are only beneficial for reducing TMJ complaints in a minority of patients; more aggressive treatment with DMARDs is generally necessary [10]. In fact, NSAIDs are effective for TMJ arthritis for one-fourth to one-third of JIA patients but primarily in oligoarticular disease. They are often considered as adjunctive or bridge therapy to more definitive interventions for TMJ disease [57]. NSAIDs are usually well tolerated. Potential side effects include gastritis, gastrointestinal bleeding, headache, increased sun sensitivity, and hepatic and/or renal dysfunction [53]. 

### 3.2. Conventional DMARDs

Conventional DMARDs include sulfasalazine, leflunomide, and methotrexate. Methotrexate is the only medication with significant evidence in the treatment of TMJ arthritis [57] and is usually first line in practice for JIA with TMJ involvement. Weekly intramuscular injection of methotrexate has been shown to decrease cartilage degeneration in rabbits with antigen-induced arthritis but failed to eliminate arthritis completely [64]. Furthermore, in a cross-sectional study, Ince et al. demonstrated that methotrexate therapy may minimize TMJ destruction in polyarticular JIA. Methotrexate is a folic acid analog that inhibits dihydrofolate reductase, leading to inhibition of purine and thymine synthesis, a reduction in T and B cell activation, and antibody formation. The dosing range is 0.5 to 1 mg/kg weekly, or 15 mg/m^2^, with a maximum dose of 25 mg weekly. It can be given by mouth or subcutaneously. Over sixty percent of patients with JIA benefit significantly, though given its slower onset of action, effects are usually not apparent until 4–6 months after initiation. Serious toxicity is uncommon, but side effects including nausea, anorexia, stomatitis, transient aminotransferase level elevation, and malaise 24 hours after administration are relatively common. Folic acid supplementation has been shown to decrease these common side effects [53]. 

### 3.3. Biologic DMARDs

Biologic DMARDs used in the treatment of TMJ inflammatory arthritis include tumor necrosis factor (TNF) inhibitors such as adalimumab, etanercept, and infliximab. These medications are usually administered systemically via subcutaneous injection (etanercept and adalimumab) or intravenous infusion (infliximab). Local therapy with intra-articular injection of infliximab has been attempted but has failed to show efficacy in improving acute or chronic synovitis, or in changing maximal incisional opening [59]. TNF inhibitors are generally given in combination with methotrexate for TMJ arthritis that is refractory to methotrexate alone. The decision on whether to initiate systemic TNF blockade when severe disease is identified or to wait until after failure of initial methotrexate is currently based on expert opinion [10]. TNF inhibition has been shown to reduce TMJ pain and improve oral function in the literature for adults, however there is not strong evidence for juvenile TMJ arthritis. Other biologic DMARDS may also be considered including tocilizumab and abatacept. Current consensus is that non-systemic JIA responds well to TNF inhibition and methotrexate while systemic JIA responds well to IL-1 and Il-6 blockade with medications such as canakinumab and tocilizumab, respectively [65]. Overall, biologic DMARDs are generally well tolerated and require minimal lab monitoring. The main adverse effect is increased risk of infection.

### 3.4. Timing of Systemic Therapy

While those with isolated TMJ arthritis may start with isolated steroid injection or irrigation, patients with polyarticular arthritis, or more systemic disease activity, benefit from antirheumatic medications. Systemic medications are generally optimized and continued until all aspects of disease including arthritis, uveitis, and systemic symptoms are well controlled. Once remission on medications is obtained, in pediatrics, treatment usually continues for at least 12–24 months before attempting to taper off, assuming the treatments are well tolerated. Recent recommendations in orthopedic literature include stopping patients’ biologic medications one dose before any planned joint replacement and waiting 14 days or until wound healing is complete until restarting the medications. New recommendations include continuing conventional DMARDs such as methotrexate during the perioperative period [62].

### 3.5. Potential Side Effects of Other Systemic Therapy

It is worth mentioning that some rheumatic disease systemic therapies, particularly bisphosphonates and corticosteroids, can be associated with TMJ disease. Bisphosphonates are potent inhibitors of osteoclastic bone resorption and are known for their use in treating osteopenia and osteoporosis but are also used in the management of chronic nonbacterial osteomyelitis (CNO, also known in the OMS literature as diffuse sclerosing osteomyelitis (DSO) or primary chronic osteomyelitis (PCO)), a rheumatic condition of inflammatory bone destruction. Jaw osteonecrosis is a potential risk of bisphosphonate use and should be considered in patients treated with bisphosphonates who present with TMJ complaints. Corticosteroids are used more widely across many rheumatic conditions as part of both acute and maintenance therapy. The side effect profile of corticosteroids will not be discussed in depth here, but it is worth noting that the risk of osteoporosis, osteopenia, and avascular necrosis is much greater when a patient is on chronic corticosteroids.

### 3.6. Systemic Therapy for Non-Rheumatic Causes of TMJ Arthritis

Traditional treatment of TMJ osteoarthritis mainly includes NSAIDs. De Souza et al [66] demonstrated equivalent pain reduction with diclofenac sodium compared with occlusal splints as well as intra-articular injections of sodium hyaluronate or corticosteroid. Research more recently has investigated oral glucosamine as an adjunctive therapy for TMJ osteoarthritis treatment. In a double-blinded randomized controlled trial conducted by Yang et al [67], oral glucosamine hydrochloride added to hyaluronate sodium injection failed to have meaningful effect on pain at month 6 post-injection but did improve pain and function at month 12, suggesting possible efficacy after prolonged use. 

Systemic treatment is not indicated for idiopathic condylar resorption (ICR), which was mentioned above as a diagnosis of exclusion and can be a mimicker of systemic rheumatic disease. While differentiating isolated TMJ JIA from ICR can be difficult, the distinction is crucial as systemic therapy is not warranted for ICR but a cornerstone of JIA management. 

## 4. Assessment of the Temporomandibular Joint in Rheumatic Disease

**Figure 3:**
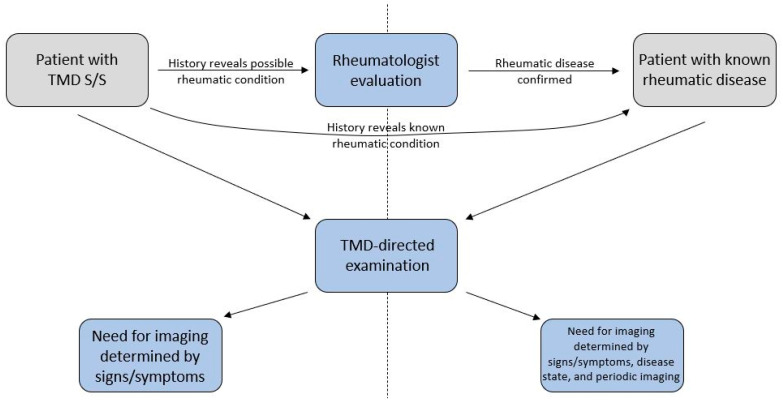
Basic framework for incorporating rheumatology referral and evaluation in patients presenting with signs and symptoms of a temporomandibular joint disorder. Patients found to have rheumatic diseases should undergo period TMJ imaging.

As noted in the Introduction, a critically important distinction between TMJ disease presentation in rheumatic diseases and non-rheumatic TMD is the delay—or even complete absence—of clinical signs and symptoms relative to anatomic destruction in rheumatologic patients. Since providers who frequently treat non-rheumatic TMD patients often do not recommend imaging until significant signs or symptoms are present, a known rheumatic diagnosis should prompt the clinician to consider earlier application of imaging modalities in this patient population (Figure 3). This may alert the provider to situations where earlier initiation of non- or minimally-invasive treatments (conventional or biologic DMARD adjustment, arthrocentesis, intra-articular medicament application, etc.) may delay further joint destruction. The relapsing/remitting nature of some of these conditions, in concert with the use of DMARDs, NSAIDs, biologics, and the associated individual patient variations in response, complicate any expected association between signs, symptoms, and imaging findings which is usually more robust in the non-rheumatic patient.

### 4.1. Patient History

For the patient without a previous rheumatic diagnosis, new signs and/or symptoms of TMD should include a broad patient history including questions regarding constitutional symptoms, pain and dysfunction of other joints, back complaints, muscle weakness, and skin/nail lesions [8]. Questions specific to vasculitides, which may occur with rheumatic conditions, can also be helpful, particularly questions about new respiratory, ophthalmologic, mucosal, or renal abnormalities.

For all patients, with or without a previous rheumatic diagnosis, a more traditional history—one more pointed at orofacial musculoskeletal disease and osteoarthritis—still remains appropriate. Questions include those regarding headaches, earaches, recent or remote trauma, parafunctional habits, and bone and cartilage diseases. Patients should specifically be asked to quantify and qualify pain, clicking, crepitus, locking, dislocation, reduced opening, stiffness, change in diet, and sense of altered occlusion. 

### 4.2. Clinical Examination

Although the TMJ clinical examination should always be comprehensive and is therefore not fundamentally different in patients with a known rheumatic disease, it does become helpful for the clinician to understand which metrics have been shown to be helpful in these patients.

The Helkimo Clinical Dysfunction Index (Di) and Helkimo Anamnestic Index (Ai) are useful metrics for assessing and monitoring such patients [68]. While the Ai is technically subjective and thus truly part of the patient’s history or subjective assessment, it is often recorded simultaneously during the clinical examination. Subjectively (Ai), the patient can be completely asymptomatic, mildly-moderately symptomatic (joint sounds, jaw fatigue, jaw stiffness), or severely symptomatic (trismus, locking, luxation, discoordination). Objectively (Di), mandibular range of motion, dysfunction with motion, and pain are measured, with significant weight being placed on end-stage pain and dysfunction (Table 2).

The Helkimo indices have been most rigorously studied in the TMJ OA population. It should be noted, however, that OA patients often present for evaluation because of pain, and thus the translatability of these results to the autoimmune population—many who either do not have pain initially or at least have a weaker association between pain and clinical and radiographic signs—should be considered cautiously. Said another way, the Helkimo index alone may underestimate the degree of damage in inflammatory rheumatic disease. Strong associations between the Helkimo index and bony changes (condylar head or fossa) but not soft tissue changes (joint space size) have been reported when using CT [69,70].

Juvenile SLE patients have been found to have significantly worse Di scores than healthy controls, with the discrepancy due primarily to TMJ dysfunction and not pain. Even more specifically, it appears that decreased laterotrusive movements may be the first signs of dysfunction in this population [29]. This has been demonstrated in the RA population as well, where worse Di scores were found to be primarily due to decreased mandibular mobility and not necessarily worse pain [71]. On the contrary, others have found that both the Ai and Di were significantly worse in RA patients than control patients, and the Helkimo indices performed significantly better at discriminating RA versus control patients than other indices [1]. As noted previously, the relapsing/remitting nature of these conditions, in concert with the use of DMARDs and NSAIDs—which frequently are not reported or controlled for as confounders in studies—complicates the association between symptoms, particularly pain, and overall cumulative TMJ damage. Accordingly, duration of autoimmune disease alone does not necessarily correlate with worsening Helkimo indices [72]. Studies generally agree that the Helkimo index as a whole helps to discriminate patients with significant arthritic disease from those without significant TMJ involvement [73,74]. A qualitative summary of studies to date finds that decreased mandibular mobility and pain on mandibular function are the most commonly reported findings. 

In addition to routine use of the Helkimo indices, international consensus guidelines have been established for orofacial examination in patients with juvenile idiopathic arthritis and can be extrapolated to the rheumatic TMD population in general [75,76]. These guidelines have resulted in a minimum recommended “short screening protocol” that includes assessment of TMJ pain in open and closed positions, mandibular deviation on opening, maximum incisal opening (MIO), frontal facial asymmetry, and facial profile. While the Helkimo indices focus more on grades of pain and dysfunction, the consensus guidelines are meant to screen for and monitor diacapitular disease activity and resulting dentofacial deformity. Monitoring in the rheumatic population will be further discussed below.

### 4.3. Imaging

#### 4.3.1. Three-Dimensional Bone Imaging

Bony destruction is reliably associated with periods of more severe disease activity. Although erosions, cortical morphology, and subcortical changes can fluctuate over time, both two-dimensional and volumetric condylar changes appear to correlate with cumulative disease activity in the joint. In RA and JIA patients, CT and MRI reveal that condylar or ramal height, condylar volume, anteroposterior length, and mediolateral width are all associated with disease severity [77], although findings are not specific to inflammatory diseases and thus cannot be used to diagnose autoimmune TMJ disease [13]. The most unifying finding in active rheumatic TMJ disease—regardless of whether the condyle, ramus, or both are affected—is asymmetry [78].

It must not be forgotten that conventional radiographs remain a reasonable screening examination in asymptomatic patients without dysfunction per the Helkimo index, with the possible exception of JIA patients. Even in this population, however, it has been suggested that condylar asymmetry on screening panoramic is specific for joint damage [79], but a concern remains for low sensitivity and reproducibility with this modality compared with three-dimensional imaging [80]. In both osteoarthritic and RA patients it has been suggested that CT may not add much to the bony changes visible on plain radiographs [81].

#### 4.3.2. Three-Dimensional Soft Tissue Imaging

MRI is the gold standard for soft tissue TMJ imaging including assessment of the articular disc, synovium, joint spaces, bone marrow, and surrounding musculature. This requires imaging protocols including fluid-sensitive (usually T2), pre-contrast T1 (usually fast spin echo, FSE), and post-contrast fat-saturated T1 sequences [82,83,84]. By far the most studied population is those with JIA, as this population is the most likely to lack signs and symptoms with significant disease activity. In a most extreme example, a study by Kellenberger et al. showed that 100% of control patients with joint effusions on MRI had pain while 0% of JIA patients with joint effusions had pain [85].

An enhancement ratio (ER) or enhancement value (EV), defined as the contrast enhancement of the superior joint space divided by that of a nearby muscle (often the longus capitis), has been described and validated in the JIA population [86,87]. Other semiquantitative MRI grading systems exist, with the OMERACT (Outcome Measures in Rheumatoid Arthritis and Clinical Trials) and EuroTMJoint (now TMJaw) research group having the most applicable systems [13]. These scoring systems evaluate inflammation (bone marrow edema, joint effusion, synovial thickening, joint enhancement) and damage (condylar flattening, erosions, and disc abnormality) and provide either a cumulative score that can be followed (OMERACT), similar to the Helkimo indices, or a progressive score (EuroTMJoint/TMJaw), similar to the Wilkes staging system. 

#### 4.3.3. Nuclear Medicine Imaging

Bone scintigraphy is a nuclear medicine examination based upon the premise that high bone turnover and/or osteoblastic activity—indicative of hyperplasia, active growth centers, or inflammatory turnover, among others—increases local uptake of radiopharmaceuticals which mimic pyrophosphate [88]. Accordingly, studies show that those with active rheumatic conditions have increased condylar uptake allowing reasonable discrimination from healthy controls and those with non-inflammatory TMJ OA, which seems to mirror discrimination by inflammatory laboratory markers (e.g., ESR, CRP) [89]. Similar findings using positron emission tomography (PET), where avidity is based upon increased glucose uptake in inflammatory environments, have been noted [90]. Nuclear medicine studies have no role in identifying TMJ OA [91].

#### 4.3.4. Ultrasonography

In theory, ultrasonography (US) seems an ideal modality to evaluate the soft tissues of the TMJ, with the TMJ being relatively superficial and US being a continuous imaging modality conducive to dynamic imaging. Unfortunately, only a few studies have attempted to objectively compare US to the current gold standard for soft tissue imaging, MRI, in autoimmune TMJ disease [11,92,93]. Although some suggest that there is at least moderate correlation between US and MRI for the assessment of synovitis in childhood arthritis [93], a recent systematic review in the JIA population could not recommend US as a standard imaging modality in these patients [94].

## 5. Interventions for Temporomandibular Joint Dysfunction in Rheumatic Disease

**Figure 4:**
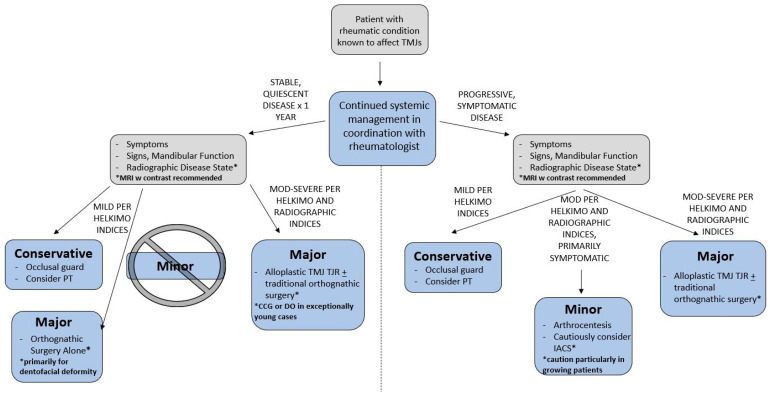
Recommended algorithm for treatment. Abbreviations: **CCG**—costochondral grafting; **DO**—distraction osteogenesis; **IACS**—intra-articular corticosteroids; mod—moderate; **PT**—physical therapy; **TJR**—total joint replacement.

Outcomes purportedly expected in the management of TMD in the rheumatic patient population should be reviewed with caution. A careful review of the available literature demonstrates that many authors reference studies involving non-rheumatic TMD patients. As the reader is already well aware, there are vast differences in presentation and outcomes in rheumatic and non-rheumatic TMD patients. Given the available evidence, an algorithm for management of rheumatic TMD patients is presented in Figure 4. Although this algorithm is based upon the TMJ Working Group’s recommendations in the JIA patient [95], less emphasis is placed on the skeletal maturity of the patient and more emphasis is placed on the disease state and degree of patient dysfunction. The central role of the systemic rheumatologic management is also highlighted by this algorithm.

### 5.1. Conservative Interventions

In non-rheumatic TMD patients, “conservative” interventions typically convey ideas of joint rest, diet alteration, occlusal guards, physical therapy, NSAIDs, and muscle relaxants. Although these certainly may also be beneficial for the rheumatic patient [96,97], the foundation of conservative management in these patients is systemic management of their inflammatory disease, as described above. That being said, self-directed physical therapy has shown effective in improving mandibular function, TMJ related pain, or both in patients with RA and AS [98,99]. In FM patients, tactile stimulation has been shown to improve sleep quality, quality of life, and TMD symptoms [100]. Low-level laser therapy for TMJ inflammatory arthritis has only been preliminarily investigated in animal models [101], and thus no conclusions regarding efficacy should be made.

Although many still propagate “occlusal equilibration” or “fixed prosthetics” for the treatment of TMD in general and TMJ OA or autoimmune diseases in particular [102,103], it should be made clear that no robust evidence supports these practices [104,105], and the senior author finds the continued use of these practices for this purpose highly misleading to patients. Although occlusal modification, including orthodontic treatment, can certainly improve facial appearance, masticatory function, and oral hygiene in these patients [106,107], it should not in any way be expected to improve rheumatic TMD.

### 5.2. Minor Procedures

#### Arthrocentesis and Intra-Articular Injection

It is well documented that arthrocentesis improves pain and dysfunction in patients with osteoarthritis, particularly Wilkes stages II, III, and IV [108,109]. Arthrocentesis with lysis and lavage alone likely improves pain and dysfunction in the rheumatic TMD population as well [110]; however, analysis is at times confounded by the fact that most rheumatic patients have traditionally also received intra-articular corticosteroid injection (IACS) at the conclusion of arthrocentesis [111]. A more recent study found that the IACS component does indeed improve the Helkimo index over arthrocentesis alone [112]. There is no question that TMJ IACS can at least temporarily improve symptoms in properly selected patients with active RA [111,113] or JIA [112], but concerns remain regarding long-term effects of IACS.

For example, multiple studies have specifically reported on the presence of heterotopic bone formation in JIA patients who have IACS, but a cause-and-effect relation has never been proven [83,114]. More recently, a retrospective review of JIA patients illustrated the complexity of the cause-and-effect relation, as the authors found that the total number of injections and time to first injection were associated with increased risk of heterotopic bone formation, yet they noted that children with more severe arthritis were likely to receive IACS [115]. Clearly, indiscriminate use of IACS should be avoided, and it should only be considered during active inflammation not responsive to medical management, preferably when confirmed by MRI [116]. Alternatively, consideration should be made for arthrocentesis with lysis and lavage *without* IACS, or with injection of hyaluronic acid [117].

More recently, intra-articular biologic injection (IAB) has been studied in the TMJ, with the first being a case report of IAB with infliximab in a patient with PsA unresponsive to both systemic infliximab and TMJ IACS [118]. Subsequent reports of IAB with infliximab in JIA patients show that although the injections appear safe, they do not affect jaw opening or improve inflammation or destruction as appreciated on MRI [119,120]. IAB with etanercept has been reported in rabbit [121] and rat [122] models of inflammatory TMJ arthritis and TMJ loading, respectively. The rabbit model showed that IAB with etanercept did not perform as well as systemic etanercept and performed no different than intra-articular saline injection. The rat model simply suggested that biochemical and biomechanical processes in the TMJ are likely driven in part by TNF-α. In conclusion, evidence to date does not support intra-articular biologic injection of the TMJs.

### 5.3. Major Procedures

#### 5.3.1. Open Arthroplasty and Associated Procedures

Synovectomy and discectomy, or possibly discectomy alone, have been shown to improve mandibular function [123] and pain [124] in patients with rheumatic TMJ disease, including RA, AS, and PsA patients. The effectiveness of these procedures should be taken into context, however, as many of these studies were performed before the application of biologic DMARDs for autoimmune rheumatic diseases. Additionally, overly aggressive attempts at or simply multiplicity of open arthroplasties may complicate eventual joint replacement, if this is foreseen in the patient’s future.

#### 5.3.2. Orthognathic Surgery

Debate continues on the stability of orthognathic surgery results in patients with resorptive TMJ processes such as inflammatory rheumatic diseases and ICR. It should also be noted that this does not treat the underlying pathology but simply masks a subset of the orofacial manifestations. The optimistic hope is that if a patient’s disease process is well controlled, the result will be stable. Unfortunately, this essentially can never be guaranteed, and therefore many “successes” end up being measured in the short term of months [125,126,127,128]. A patient with a process defined by condylar resorption electing to undergo orthognathic surgery alone must absolutely be informed that relapse is expected, TMJ pain and dysfunction are not expected to resolve, and only TMJ TJR will predictably result in long-term stability [129]. Thus, the patient best suited for orthognathic surgery alone is one with stably quiescent disease with relatively mild deformities.

Condylotomy—which has evolved to its current day form of essentially a vertical ramus osteotomy—has been documented as a treatment in active inflammatory TMD [103], but this represents a lack of understanding of the disease process and should not be performed.

#### 5.3.3. Distraction Osteogenesis

Distraction osteogenesis (DO) of the mandibular rami has been reported in JIA patients. As would be expected for a treatment aimed primarily at altering the dentofacial abnormality without addressing the TMJ disease process itself, facial appearance and occlusal relationship were improved while long-term pain, mandibular mobility, and TMJ signs had either mixed results or continued progression [130,131]. It should also be noted that inclusion criteria in the only prospective study to date were unilateral TMJ involvement, inactive disease, and TMJs with “clinical and subjective good function” preoperatively [130]. Therefore, similar to the potential orthognathic patient, patients with a process defined by condylar resorption electing to undergo DO alone must absolutely be informed that relapse is expected, TMJ pain and dysfunction are not expected to resolve, and only TMJ TJR will predictably result in long-term stability.

#### 5.3.4. Total Joint Replacement

Although historically costochondral grafting (CCG) has been performed in patients with rheumatic TMD [132,133,134], and although debate continues on the application of autogenous or alloplastic procedures for TMJ TJR in non-rheumatic end-stage joint disease, the senior author agrees with the idea that inflammatory TMJ destruction is best treated with alloplastic methods [135].

Guidelines have been put forth to guide physicians when prosthetic TMJ TJR may be appropriate, including in inflammatory joint disease [136]. Not surprisingly, the superiority of alloplastic TMJ TJR in non-rheumatic end-stage joint disease patients has been found to translate to the autoimmune population as well [137]. Outcomes of alloplastic TMJ TJR in RA, PsA, AS, SSc, and JIA patients have been reported, showing consistent improvement in associated pain and dysfunction [138,139,140,141,142,143,144,145,146,147]. The literature nearly unanimously suggests that patients with appropriate indications for TMJ TJR have seen improved, durable outcomes.

A legitimate concern in open surgery—particularly those involving alloplastic implantation—is the immunosuppressive therapies that many patients will be taking, particularly those patients with disease severe enough to require such surgery [116]. Studies of TMJ TJR often do not comment on perioperative medication management, although this is vitally important to success. Although developed with the American Association of Hip and Knee Surgeons (AAHKS), the ACR has published perioperative guidelines for management of antirheumatic medications in those undergoing arthroplasty [148]. As mentioned previously, conventional DMARDs should generally be continued through surgery while surgery should occur at the end of biologic DMARD dosing cycles, and the biologic should not be resumed until 14 or more days post-operatively (assuming no post-operative infectious or wound healing complications). 

## 6. Monitoring of the Rheumatic Patient with Temporomandibular Joint Disease

There are minimal evidence-based or consensus guidelines for monitoring in rheumatic patients with TMD, with most available data pertaining to the JIA population. Consensus assessment methods were reached by the Temporomandibular Joint Juvenile Arthritis (TMJaw) Working Group for monitoring of TMJ arthritis and involvement in JIA patients in 2019 [95]. These include MRI with contrast, 3D scans (which may include CBCT or medical grade CT as appropriate), clinical examination, and patient-reported outcome measures. Consensus could not be reached to recommend the use of MRI without contrast, plain radiographs, or ultrasound in the monitoring of TMJ arthritis in these patients. The TMJaw group also proposed a clinical evaluation protocol for regular assessment of the TMJ joint in patients with JIA, which is applicable both for screening as well as following patients with a history of TMJ arthritis [75,76]. As discussed above, the components of the exam allow for a quick assessment of pain, range of motion, and dentofacial deformity and asymmetry, which when followed over time can assist in detecting subtle changes indicative of active or progressive disease. However, as previously stated, given the potential for active, erosive TMJ arthritis in asymptomatic or minimally symptomatic patients, there is also a need for imaging to both evaluate for initial disease, as well as to follow the course of TMJ arthritis during and after treatment. This is the case when following up after either TMJ arthrocentesis or initiation of systemic rheumatic medications. Given MRI with contrast is the gold standard for active synovitis, monitoring 6 months after a treatment is initiated or changed with an MRI is the most accurate for assessing whether there is ongoing disease activity that would warrant additional measures. 

A survey of academic American OMS practice patterns in managing and monitoring JIA patients suggests that once inflammatory arthritic patients are deemed to be in remission, most are monitored at 6 to 12 month intervals [61]. However, this study also revealed that the average OMS often relies more on symptoms and plain radiography rather than MRI when following this patient population. This highlights the potential benefit of ongoing discussions between rheumatology and OMS to determine the best imaging modality for individual patients. 

**Figure 5:**
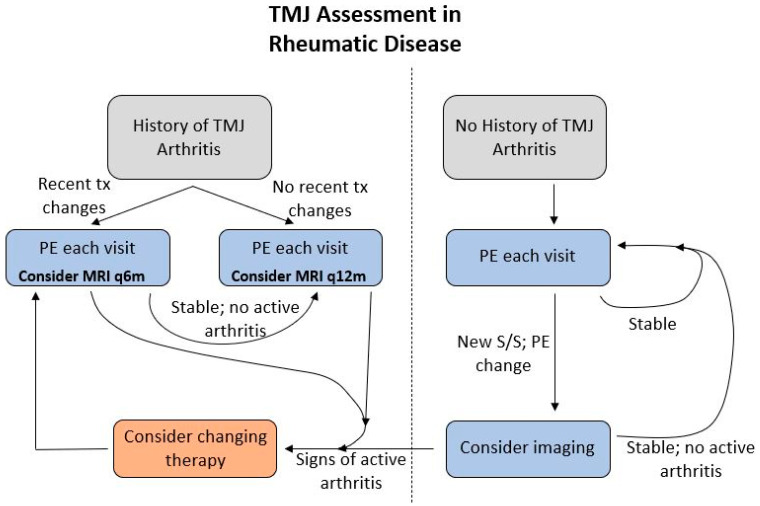
Recommended monitoring protocol. Abbreviations: **q6m**—every 6 months; **q12m**—every 12 months; **PE**—physical exam; **S/S**—signs and symptoms; **tx**—treatment.

With regard to monitoring for disease activity and its effect on surgical treatment decisions, the TMJaw group recommends that a lack of progression over one year combined with contrasted MRI confirmation of quiescent disease serves as reasonable evidence to proceed with autologous reconstruction (e.g., costochondral grafting and/or orthognathic surgery). The unpredictability of the disease process, particularly in younger patients, should be considered however when deciding on surgical intervention. A suggested monitoring protocol is presented in Figure 5.

## Figures and Tables

**Table 1:** Abbreviated summary of diagnostic criteria for various rheumatic diseases affecting the TMJ. Criteria provided have been commonly used for clinical and/or research purposes. Abbreviations: **ACR**—American College of Rheumatology; **ANA**—anti-nuclear antibodies; **ARA**—American Rheumatism Association (predecessor of the ACR); **ASAS**—Assessment of Spondyloarthritis International Society; **axSpA**—axial spondyloarthritis; **CASPAR**—Classification of Psoriatic Arthritis Study; **CCP**—cyclic citrullinated peptide; **CRP**—C-reactive protein; **ESR**—erythrocyte sedimentation rate; **EULAR**—European League Against Rheumatism; **FM**—fibromyalgia; **ILAR**—International League of Associations for Rheumatology; **JIA**—juvenile idiopathic arthritis; **PsA**—psoriatic arthritis; **RA**—rheumatoid arthritis; **RF**—rheumatoid factors; **SLE**—systemic lupus erythematosus; **SSc**—systemic sclerosis; **SSS**—symptom severity scale; **WPI**—widespread pain index.

Condition	Abbreviated Diagnostic Criteria	
JIA	**ILAR/EULAR (2001)** Arthritis 6+ weeks Age < 16 years at diagnosis No other identifiable cause	
SSc	**ACR/EULAR (2013)** *Each item is weighted with total score ≥ 9 indicative of definite SSc* Skin thickening of fingers Fingertip lesions Telangiectasias Abnormal nailfold capillaries Pulmonary arterial hypertension and/or interstitial lung disease Raynaud phenomenon Anti-centromere, -topoisomerase I, or -RNA polymerase III antibodies	
RA	**ARA (1987)** *4+ of the following* Morning stiffness for 6+ weeks Arthritis, 3+ joints, for 6+ weeks Arthritis, hands, for 6+ weeks Symmetric arthritis for 6+ weeks Rheumatoid nodules RF+ Radiographic change	**ACR/EULAR (2010)** Synovitis, 1+ joint Absence of alternative diagnosis *AND* Score of 6+ out of the following, which are weighted: Number of involved joints RF+ or anti-CCP+ Elevated ESR or CRP Symptoms 6+ weeks
SLE	**ACR (1997)** 4+ of 11 criteria including mucocutaneous and major organ clinical criteria and immunologic laboratory criteria	**ACR/EULAR (2019)** ANA+ or equivalent At least one clinical criterion Additional clinical or immunologic criteria are weighted and are additive towards a final score (≥10 = SLE)
axSpA	**ASAS (2009)** 3+ months of back pain with onset prior to 45 years of age *IF* sacroiliitis on imaging, 1+ SpA feature is required *IF* no sacroiliitis on imaging, HLA-B27+ and 2+ SpA features are required	
PsA	**CASPAR (2006)** *3+ points from weighted items of the following* Skin psoriasis Nail lesions Dactylitis Negative RF Juxta-articular bone formation	
FM	**ACR (1990)** Widespread, chronic pain 11+ of 18 tender points No other identifiable cause	**ACR (2010)** WPI ≥ 7 + SSS ≥ 5 *OR* WPI ≥ 3 + SSS ≥ 9 Symptoms 3+ months No other identifiable cause

**Table 2:** Helkimo clinical dysfunction index (Di). The maximum score recorded from each domain is added to determine the total clinical dysfunction index score. Abbreviations: **MIO**—maximum incisal opening; mm—millimeter.

Domain	Criteria	Score
Mandibular Mobility	MIO > 40 mm, excursions > 7 mm MIO > 30 mm, excursions > 3 mm MIO ≤ 30 mm, excursions ≤ 3 mm	0 1 5
TMJ Dysfunction	No sounds/deviation on opening Sounds and/or deviation > 2 mm Locking and/or luxation	0 1 5
Muscle Pain	No muscle pain Pain on palpation at 1–3 sites Pain on palpation at 4+ sites	0 1 5
TMJ Pain	No tenderness to palpation Lateral (superficial) TMJ pain Posterior (deep) TMJ pain	0 1 5
Mandibular Movement Pain	No pain Pain with 1 movement Pain with 2+ movements	0 1 5
Di 0: 0 points—absence of dysfunction		
Di I: 1–4 points—mild dysfunction		
Di II: 5–9 points—moderate dysfunction		
Di III: 10–25 points—severe dysfunction		

## References

[1] Da Cunha S.C., Nogueira R.V., Duarte A.P., Vasconcelos B.C., de Almeida R.A. (2007). Analysis of helkimo and craniomandibular indexes for temporomandibular disorder diagnosis on rheumatoid arthritis patients. Braz. J. Otorhinolaryngol..

[2] Helmick C.G., Felson D.T., Lawrence R.C., Gabriel S., Hirsch R., Kwoh C.K., Liang M.H., Kremers H.M., Mayes M.D., Merkel P.A. (2008). National Arthritis Data Workgroup. Estimates of the prevalence of arthritis and other rheumatic conditions in the United States: Part I. Arthritis Rheum..

[3] Centers for Disease Control and Prevention (2017). Arthritis in America. https://www.cdc.gov/vitalsigns/arthritis/index.html.

[4] World Health Organization Chronic Rheumatic Conditions. https://www.who.int/chp/topics/rheumatic/en/.

[5] Wilkes C.H. (1989). Internal derangements of the temporomandibular joint. Pathological variations. Arch. Otolaryngol. Head Neck Surg..

[6] Hirahara N., Kaneda T., Muraoka H., Fukuda T., Ito K., Kawashima Y. (2017). Characteristic magnetic resonance imaging findings in rheumatoid arthritis of the temporomandibular joint: Focus on abnormal bone marrow sgnal of the mandibular condyle, pannus, and lymph node swelling in the parotid glands. J. Oral Maxillofac. Surg..

[7] Scutellari P.N., Orzincolo C., Ceruti S. (1993). L’articolazione temporo-mandibolare nelle condizioni patologiche: Artrite reumatoide e spondiloartriti sieronegative. (The temporo-mandibular joint in pathologic conditions: Rheumatoid arthritis and seronegative spondyloarthritis). Radiol. Med..

[8] Sidebottom A.J., Salha R. (2013). Management of the temporomandibular joint in rheumatoid disorders. Br. J. Oral Maxillofac. Surg..

[9] Petty R.E., Southwood T.R., Manners P., Baum J., Glass D.N., Goldenberg J., He X., Maldonado-Cocco J., Orozco-Alcala J., Prieur A.M. (2004). International League of Associations for Rheumatology. International League of Associations for Rheumatology classification of juvenile idiopathic arthritis: Second revision, Edmonton, 2001. J. Rheumatol..

[10] Veldhuis E.C., Veldhuis A.H., Koudstaal M.J. (2014). Treatment management of children with juvenile idiopathic arthritis with temporomandibular joint involvement: A systematic review. Oral Surg. Oral Med. Oral Pathol. Oral Radiol..

[11] Weiss P.F., Arabshahi B., Johnson A., Bilaniuk L.T., Zarnow D., Cahill A.M., Feudtner C., Cron R.Q. (2008). High prevalence of temporomandibular joint arthritis at disease onset in children with juvenile idiopathic arthritis, as detected by magnetic resonance imaging but not by ultrasound. Arthritis Rheum..

[12] Melchiorre D., Falcini F., Kaloudi O., Bandinelli F., Nacci F., Matucci Cerinic M. (2010). Sonographic evaluation of the temporomandibular joints in juvenile idiopathic arthritis. J. Ultrasound.

[13] Kellenberger C.J., Junhasavasdikul T., Tolend M., Doria A.S. (2018). Temporomandibular joint atlas for detection and grading of juvenile idiopathic arthritis involvement by magnetic resonance imaging. Pediatr. Radiol..

[14] Seifert M.H., Steigerwald J.C., Cliff M.M. (1975). Bone resorption of the mandible in progressive systemic sclerosis. Arthritis Rheum..

[15] Osial T.A., Avakian A., Sassouni V., Agarwal A., Medsger T.A., Rodnan G.P. (1981). Resorption of the mandibular condyles and coronoid processes in progressive systemic sclerosis (scleroderma). Arthritis Rheum..

[16] Ferreira E.L., Christmann R.B., Borba E.F., Borges C.T., Siqueira J.T., Bonfa E. (2010). Mandibular function is severely impaired in systemic sclerosis patients. J. Orofac. Pain.

[17] Aliko A., Ciancaglini R., Alushi A., Tafaj A., Ruci D. (2011). Temporomandibular joint involvement in rheumatoid arthritis, systemic lupus erythematosus and systemic sclerosis. Int. J. Oral Maxillofac. Surg..

[18] Crincoli V., Fatone L., Fanelli M., Rotolo R.P., Chialà A., Favia G., Lapadula G. (2016). Orofacial Manifestations and Temporomandibular Disorders of Systemic Scleroderma: An Observational Study. Int. J. Mol. Sci..

[19] Bessa-Nogueira R.V., Vasconcelos B.C., Duarte A.P., Góes P.S., Bezerra T.P. (2008). Targeted assessment of the temporomandibular joint in patients with rheumatoid arthritis. J. Oral Maxillofac. Surg..

[20] Aceves-Avila F.J., Chávez-López M., Chavira-González J.R., Ramos-Remus C. (2013). Temporomandibular joint dysfunction in various rheumatic diseases. Reumatismo.

[21] Cordeiro P.C., Guimaraes J.P., de Souza V.A., Dias I.M., Silva J.N., Devito K.L., Bonato L.L. (2016). Temporomandibular joint involvement in rheumatoid arthritis patients: Association between clinical and tomographic data. Acta Odontol. Latinoam..

[22] Celiker R., Gökçe-Kutsal Y., Eryilmaz M. (1995). Temporomandibular joint involvement in rheumatoid arthritis. Relationship with disease activity. Scand. J. Rheumatol..

[23] Yoshida A., Higuchi Y., Kondo M., Tabata O., Ohishi M. (1998). Range of motion of the temporomandibular joint in rheumatoid arthritis: Relationship to the severity of disease. Cranio.

[24] Mortazavi N., Babaei M., Babaee N., Kazemi H.H., Mortazavi R., Mostafazadeh A. (2018). Evaluation of the prevalence of temporomandibular joint involvement in rheumatoid arthritis using research diagnostic criteria for temporomandibular disorders. J. Dent..

[25] Redlund-Johnell I. (1987). Severe rheumatoid arthritis of the temporomandibular joints and its coincidence with severe rheumatoid arthritis of the cervical spine. Scand. J. Rheumatol..

[26] Ozcan I., Ozcan K.M., Keskin D., Bahar S., Boyacigil S., Dere H. (2008). Temporomandibular joint involvement in rheumatoid arthritis: Correlation of clinical, laboratory and magnetic resonance imaging findings. B ENT.

[27] Crincoli V., Piancino M.G., Iannone F., Errede M., Di Comite M. (2020). Temporomandibular disorders and oral features in systemic lupus erythematosus patients: An observational study of symptoms and signs. Int. J. Med. Sci..

[28] Jonsson R., Lindvall A.M., Nyberg G. (1983). Temporomandibular joint involvement in systemic lupus erythematosus. Arthritis Rheum..

[29] Fernandes E.G., Savioli C., Siqueira J.T., Silva C.A. (2007). Oral health and the masticatory system in juvenile systemic lupus erythematosus. Lupus.

[30] Grinin V.M., Maksimovskiĭ I.M., Nasonova V.A. (1999). Asepticheskiĭ nekroz visochno-nizhnecheliustnogo sustava pri sistemnoĭ krasnoĭ volchanke [Aseptic necrosis of the temporomandibular joint in systemic lupus erythematosus]. Stomatologiia.

[31] Grinin V.M. (1995). Kontseptsiia patogeneza okkliuzionnykh narusheniĭ pri zabolevaniiakh visochno-nizhnecheliustnogo sustava [The concept of the pathogenesis of occlusive disorders in diseases of the temporomandibular joint]. Stomatologiia.

[32] Grinin V.M., Nasonova V.A., Maksimovskiĭ I.M., Ternovoĭ S.K., Sinitsyn V.E., Smirnov A.V. (2000). Differentsial’naia diagnostika avaskuliarnogo nekroza visochno-nizhnecheliustnogo sustava pri sistemnoĭ krasnoĭ volchanke [The differential diagnosis of avascular necrosis of the temporomandibular joint in systemic lupus erythematosus]. Stomatologiia.

[33] Szántó D., Bohátka L., Csokonay L., Schiefner G., Boross G., Jáger M., Barzó P. (1986). A capitulum mandibulae avascularis necrosisa systemás lupus erythematosusban [Avascular necrosis of the mandibular condyle in systemic lupus erythematosus]. Orv. Hetil..

[34] Costantino F., Zeboulon N., Said-Nahal R., Breban M. (2017). Radiographic sacroiliitis develops predictably over time in a cohort of familial spondyloarthritis followed longitudinally. Rheumatology.

[35] Mathieu A., Paladini F., Vacca A., Cauli A., Fiorillo M.T., Sorrentino R. (2009). The interplay between the geographic distribution of HLA-B27 alleles and their role in infectious and autoimmune diseases: A unifying hypothesis. Autoimmun. Rev..

[36] Davidson C., Wojtulewski J.A., Bacon P.A., Winstock D. (1975). Temporo-mandibular joint disease in ankylosing spondylitis. Ann. Rheum. Dis..

[37] Li J.M., Zhang X.W., Zhang Y., Li Y.H., An J.G., Xiao E., Yan Y.B. (2013). Ankylosing spondylitis associated with bilateral ankylosis of the temporomandibular joint. Oral Surg. Oral Med. Oral Pathol. Oral Radiol..

[38] Koorbusch G.F., Zeitler D.L., Fotos P.G., Doss J.B. (1991). Psoriatic arthritis of the temporomandibular joints with ankylosis. Literature review and case reports. Oral Surg. Oral Med. Oral Pathol..

[39] Alinaghi F., Calov M., Kristensen L.E., Gladman D.D., Coates L.C., Jullien D., Gottlieb A.B., Gisondi P., Wu J.J., Thyssen J.P. (2019). Prevalence of psoriatic arthritis in patients with psoriasis: A systematic review and meta-analysis of observational and clinical studies. J. Am. Acad. Dermatol..

[40] Wright V., Moll J.M. (1971). Psoriatic arthritis. Bull. Rheum. Dis..

[41] Farronato G., Garagiola U., Carletti V., Cressoni P., Bellintani C. (2010). Psoriatic arthritis: Temporomandibular joint involvement as the first articular phenomenon. Quintessence Int..

[42] Dervis E., Dervis E. (2005). The prevalence of temporomandibular disorders in patients with psoriasis with or without psoriatic arthritis. J. Oral Rehabil..

[43] Badel T., Savić Pavičin I., Krapac L., Zadravec D., Rosić D. (2014). Psoriatic arthritis and temporomandibular joint involvement—Literature review with a reported case. Acta Dermatovenerol. Croat..

[44] Okeson J. (2019). Management of Temporomandibular Disorders and Occlusion.

[45] Tanaka E., Detamore M.S., Mercuri L.G. (2008). Degenerative disorders of the temporomandibular joint: Etiology, diagnosis, and treatment. J. Dent. Res..

[46] Matsumoto R., Ioi H., Goto T.K., Hara A., Nakata S., Nakasima A., Counts A.L. (2010). Relationship between the unilateral TMJ osteoarthritis/osteoarthrosis, mandibular asymmetry and the EMG activity of the masticatory muscles: A retrospective study. J. Oral Rehabil..

[47] Altman R., Asch E., Bloch D., Bole G., Borenstein D., Brandt K., Christy W., Cooke T.D., Greenwald R., Hochberg M. (1986). Development of criteria for the classification and reporting of osteoarthritis. Classification of osteoarthritis of the knee. Diagnostic and Therapeutic Criteria Committee of the American Rheumatism Association. Arthritis Rheum..

[48] Altman R., Alarcón G., Appelrouth D., Bloch D., Borenstein D., Brandt K., Brown C., Cooke T.D., Daniel W., Feldman D. (1991). The American College of Rheumatology criteria for the classification and reporting of osteoarthritis of the hip. Arthritis Rheum..

[49] Balasubramaniam R., Laudenbach J.M., Stoopler E.T. (2007). Fibromyalgia: An update for oral health care providers. Oral Surg. Oral Med. Oral Pathol. Oral Radiol. Endodontol..

[50] Ayouni I., Chebbi R., Hela Z., Dhidah M. (2019). Comorbidity between fibromyalgia and temporomandibular disorders: A systematic review. Oral Surg. Oral Med. Oral Pathol. Oral Radiol..

[51] Arnett G.W., Milam S.B., Gottesman L. (1996). Progressive mandibular retrusion—Idiopathic condylar resorption: Part I. Am. J. Orthod. Dentofac. Orthop..

[52] Abubaker A.O., Raslan W.F., Sotereanos G.C. (1993). Estrogen and progesterone receptors in temporomandibular joint discs of symptomatic and asymptomatic persons: A preliminary study. J. Oral Maxillofac. Surg..

[53] Abramowicz S., Kim S., Prahalad S., Chouinard A.F., Kaban L.B. (2016). Juvenile arthritis: Current concepts in terminology, etiopathogenesis, diagnosis, and management. Int. J. Oral Maxillofac. Surg..

[54] Huang Y.L., Pogrel M.A., Kaban L.B. (1997). Diagnosis and management of condylar resorption. J. Oral Maxillofac. Surg..

[55] Alsabban L., Amarista F.J., Mercuri L.G., Perez D. (2018). Idiopathic condylar resorption: A survey and review of the literature. J. Oral Maxillofac. Surg..

[56] Stoustrup P., Twilt M., Herlin T. (2020). Systemic treatment for temporomandibular joint arthritis in juvenile idiopathic arthritis. J. Rheumatol..

[57] Carrasco R. (2015). Juvenile idiopathic arthritis overview and involvement of the temporomandibular joint. Oral Maxillofac. Surg. Clin. N. Am..

[58] Bollhalder A., Patcas R., Eichenberger M., Muller L., Schroeder-Kohler S., Saurenmann R.K., Kellenberger C.J. (2020). Magnetic resonance imaging followup of temporomandibular Joint inflammation, deformation, and mandibular growth in juvenile idiopathic arthritis patients receiving systemic treatment. J. Rheumatol..

[59] Stoll M.L., Kau C.H., Waite P.D., Cron R.Q. (2018). Temporomandibular joint arthritis in juvenile idiopathic arthritis, now what?. Pediatr. Rheumatol..

[60] Foeldvari I., Tzaribachev N., Cron R.Q. (2014). Results of a multinational survey regarding the diagnosis and treatment of temporomandibular joint involvement in juvenile idiopathic arthritis. Pediatr. Rheumatol..

[61] Kinard B.E., Abramowicz S. (2017). Juvenile idiopathic arthritis practice patterns among oral and maxillofacial surgeons. J. Oral Maxillofac. Surg..

[62] Granquist E.F. (2018). Treatment of the temporomandibular Joint in a child with juvenile idiopathic arthritis. Oral Maxillofac. Surg. Clin. N. Am..

[63] Wang X.D., Zhang J.N., Gan Y.H., Zhou Y.H. (2015). Current understanding of pathogenesis and treatment of TMJ osteoarthritis. J. Dent. Res..

[64] Rafayelyan S., Meyer P., Radlanski R.J., Minden K., Jost-Brinkmann P.G., Präger T.M. (2015). Effect of methotrexate upon antigen-induced arthritis of the rabbit temporomandibular joint. J. Oral Pathol. Med..

[65] Nilbo P., Pruunsild C., Voog-Oras U., Nikopensius T., Jagomagi T., Saag M. (2016). Contemporary management of TMJ involvement in JIA patients and its orofacial consequences. EPMA J..

[66] De Souza R.F., Lovato da Silva C.H., Nasser M., Fedorowicz Z., Al-Muharrqai M.A. (2012). Interventions for managing temporomandibular joint osteoarthritis. Cochrane Database Syst. Rev..

[67] Yang W., Lie W., Miao C., Sun H., Li L., Li C. (2018). Oral glucosamine hydrochloride combined with hyaluronate sodium intra-articular injection for temporomandibular joint osteoarthritis: A double blind randomized controlled trial. J. Oral Maxillofac. Surg..

[68] Helkimo M. (1974). Studies on function and dysfunction of the masticatory system. II. Index for anamnestic and clinical dysfunction and occlusal state. Sven Tandlak. Tidskr..

[69] Su N., Liu Y., Yang X., Luo Z., Shi Z. (2014). Correlation between bony changes measured with cone beam computed tomography and clinical dysfunction index in patients with temporomandibular joint osteoarthritis. J. Craniomaxillofac. Surg..

[70] Hiltunen K., Vehkalahti M.M., Peltola J.S., Ainamo A. (2002). A 5-year follow-up of occlusal status and radiographic findings in mandibular condyles of the elderly. Int. J. Prosthodont..

[71] Gleissner C., Kaesser U., Dehne F., Bolten W.W., Willershausen B. (2003). Temporomandibular joint function in patients with longstanding rheumatoid arthritis—I. Role of periodontal status and prosthetic care—A clinical study. Eur. J. Med. Res..

[72] Witulski S., Vogl T.J., Rehart S., Ottl P. (2014). Evaluation of the TMJ by means of clinical TMD examination and MRI diagnostics in patients with rheumatoid arthritis. Biomed. Res. Int..

[73] Capurso U., De Michelis B., Giaretta Agosti G., Lepore L. (1989). Compromissione funzionale dell’apparato masticatorio nell’artrite reumatoide giovanile [Compromised function of the masticatory apparatus in juvenile rheumatoid arthritis]. Minerva Ortognatod..

[74] Capurso U., Scutellari P.N., Orzincolo C., Calura G. (1989). La compromissione dell’articolazione temporo-mandibolare nell’artrite reumatoide [Involvement of the temporomandibular joint in rheumatoid arthritis]. Radiol. Med..

[75] Stoustrup P., Herlin T., Spiegel L., Rahimi H., Koos B., Pedersen T.K., Twilt M. (2020). Temporomandibular joint juvenile arthritis working group. standardizing the clinical orofacial examination in juvenile idiopathic arthritis: An interdisciplinary, consensus-based, short screening protocol. J. Rheumatol..

[76] Stoustrup P., Twilt M., Spiegel L., Kristensen K.D., Koos B., Pedersen T.K., Küseler A., Cron R.Q., Abramowicz S., Verna C. (2017). EuroTM joint Research Network. Clinical orofacial examination in juvenile idiopathic arthritis: International consensus-based recommendations for monitoring patients in clinical practice and research studies. J. Rheumatol..

[77] Youssef Mohamed M.M., Dahaba M.M., Farid M.M., Ali Elsayed A.M. (2020). Radiographic changes in TMJ in relation to serology and disease activity in RA patients. Dentomaxillofac. Radiol..

[78] Koos B., Gassling V., Bott S., Tzaribachev N., Godt A. (2014). Pathological changes in the TMJ and the length of the ramus in patients with confirmed juvenile idiopathic arthritis. J. Craniomaxillofac. Surg..

[79] Piancino M.G., Cannavale R., Dalmasso P., Tonni I., Filipello F., Perillo L., Cattalini M., Meini A. (2015). Condylar asymmetry in patients with juvenile idiopathic arthritis: Could it be a sign of a possible temporomandibular joints involvement?. Semin. Arthritis Rheum..

[80] Poveda-Roda R., Bagan J., Carbonell E., Margaix M. (2015). Diagnostic validity (sensitivity and specificity) of panoramic X-rays in osteoarthrosis of the temporomandibular joint. Cranio.

[81] Modgil R., Arora K.S., Sharma A., Negi L.S., Mohapatra S., Pareek S. (2019). TMJ arthritis imaging: Conventional radiograph vs. CT scan—Is CT actually needed?. Curr. Rheumatol. Rev..

[82] Kellenberger C.J., Abramowicz S., Arvidsson L.Z., Kirkhus E., Tzaribachev N., Larheim T.A. (2018). Recommendations for a standard magnetic resonance imaging protocol of temporomandibular joints in juvenile idiopathic arthritis. J. Oral Maxillofac. Surg..

[83] Lochbühler N., Saurenmann R.K., Müller L., Kellenberger C.J. (2015). Magnetic resonance imaging assessment of temporomandibular Joint Involvement and mandibular growth following corticosteroid injection in juvenile idiopathic arthritis. J. Rheumatol..

[84] Miller E., Inarejos Clemente E.J., Tzaribachev N., Guleria S., Tolend M., Meyers A.B., von Kalle T., Stimec J., Koos B., Appenzeller S. (2018). Imaging of temporomandibular joint abnormalities in juvenile idiopathic arthritis with a focus on developing a magnetic resonance imaging protocol. Pediatr. Radiol..

[85] Kellenberger C.J., Bucheli J., Schroeder-Kohler S., Saurenmann R.K., Colombo V., Ettlin D.A. (2019). Temporomandibular joint magnetic resonance imaging findings in adolescents with anterior disk displacement compared to those with juvenile idiopathic arthritis. J. Oral Rehabil..

[86] Resnick C.M., Vakilian P.M., Breen M., Zurakowski D., Caruso P., Henderson L., Nigrovic P.A., Kaban L.B., Peacock Z.S. (2016). Quantifying temporomandibular joint synovitis in children with juvenile idiopathic arthritis. Arthritis Care Res..

[87] Buch K., Peacock Z.S., Resnick C.M., Rothermel H., Kaban L.B., Caruso P. (2020). Regional differences in temporomandibular joint inflammation in patients with juvenile idiopathic arthritis: A dynamic post-contrast magnetic resonance imaging study. Int. J. Oral Maxillofac. Surg..

[88] Epstein J.B., Rea A., Chahal O. (2002). The use of bone scintigraphy in temporomandibular joint disorders. Oral Dis..

[89] Shim J.S., Kim C., Ryu J.J., Choi S.J. (2020). Correlation between TM joint disease and rheumatic diseases detected on bone scintigraphy and clinical factors. Sci. Rep..

[90] Mupparapu M., Oak S., Chang Y.C., Alavi A. (2019). Conventional and functional imaging in the evaluation of temporomandibular joint rheumatoid arthritis: A systematic review. Quintessence Int..

[91] Kang J.H., An Y.S., Park S.H., Song S.I. (2018). Influences of age and sex on the validity of bone scintigraphy for the diagnosis of temporomandibular joint osteoarthritis. Int. J. Oral Maxillofac. Surg..

[92] Müller L., Kellenberger C.J., Cannizzaro E., Ettlin D., Schraner T., Bolt I.B., Peltomäki T., Saurenmann R.K. (2009). Early diagnosis of temporomandibular joint involvement in juvenile idiopathic arthritis: A pilot study comparing clinical examination and ultrasound to magnetic resonance imaging. Rheumatology.

[93] Kirkhus E., Gunderson R.B., Smith H.J., Flatø B., Hetlevik S.O., Larheim T.A., Arvidsson L.Z. (2016). Temporomandibular joint involvement in childhood arthritis: Comparison of ultrasonography-assessed capsular width and MRI-assessed synovitis. Dentomaxillofac. Radiol..

[94] Hechler B.L., Phero J.A., Van Mater H., Matthews N.S. (2018). Ultrasound versus magnetic resonance imaging of the temporomandibular joint in juvenile idiopathic arthritis: A systematic review. Int. J. Oral Maxillofac. Surg..

[95] Resnick C.M., Frid P., Norholt S.E., Stoustrup P., Peacock Z.S., Kaban L.B., Pedersen T.K., Abramowicz S., Temporomandibular Joint Juvenile Arthritis (TMJaw) working group (2019). An algorithm for management of dentofacial deformity resulting from juvenile idiopathic arthritis: Results of a multinational consensus conference. J. Oral Maxillofac. Surg..

[96] Isola G., Ramaglia L., Cordasco G., Lucchese A., Fiorillo L., Matarese G. (2017). The effect of a functional appliance in the management of temporomandibular joint disorders in patients with juvenile idiopathic arthritis. Minerva Stomatol..

[97] Stoustrup P., Küseler A., Kristensen K.D., Herlin T., Pedersen T.K. (2013). Orthopaedic splint treatment can reduce mandibular asymmetry caused by unilateral temporomandibular involvement in juvenile idiopathic arthritis. Eur. J. Orthod..

[98] Tegelberg A., Kopp S. (1988). Short-term effect of physical training on temporomandibular joint disorder in individuals with rheumatoid arthritis and ankylosing spondylitis. Acta Odontol. Scand..

[99] Tegelberg A., Kopp S. (1996). A 3-year follow-up of temporomandibular disorders in rheumatoid arthritis and ankylosing spondylitis. Acta Odontol. Scand..

[100] Adiels A.M., Helkimo M., Magnusson T. (2005). Tactile stimulation as a complementary treatment of temporomandibular disorders in patients with fibromyalgia syndrome. A pilot study. Swed. Dent. J..

[101] Khozeimeh F., Moghareabed A., Allameh M., Baradaran S. (2015). Comparative evaluation of low-level laser and systemic steroid therapy in adjuvant-enhanced arthritis of rat temporomandibular joint: A histological study. Dent. Res. J..

[102] Shoohanizad E., Garajei A., Enamzadeh A., Yari A. (2019). Nonsurgical management of temporomandibular joint autoimmune disorders. AIMS Public Health.

[103] Puricelli E., Corsetti A., Tavares J.G., Luchi G.H. (2013). Clinical-surgical treatment of temporomandibular joint disorder in a psoriatic arthritis patient. Head Face Med..

[104] List T., Axelsson S. (2010). Management of TMD: Evidence from systematic reviews and meta-analyses. J. Oral Rehabil..

[105] Koh H., Robinson P.G. (2003). Occlusal adjustment for treating and preventing temporomandibular joint disorders. Cochrane Database Syst. Rev..

[106] Kuroda S., Kuroda Y., Tomita Y., Tanaka E. (2012). Long-term stability of conservative orthodontic treatment in a patient with rheumatoid arthritis and severe condylar resorption. Am. J. Orthod. Dentofac. Orthop..

[107] Ferri J., Potier J., Maes J.M., Rakotomalala H., Lauwers L., Cotelle M., Nicot R. (2018). Temporomandibular joint arthritis: Clinical, orthodontic, orthopaedic and surgical approaches. Int. Orthod..

[108] Leibur E., Jagur O., Voog-Oras Ü. (2015). Temporomandibular joint arthrocentesis for the treatment of osteoarthritis. Stomatologija.

[109] Nitzan D.W., Price A. (2001). The use of arthrocentesis for the treatment of osteoarthritic temporomandibular joints. J. Oral Maxillofac. Surg..

[110] Gynther G.W., Holmlund A.B. (1998). Efficacy of arthroscopic lysis and lavage in patients with temporomandibular joint symptoms associated with generalized osteoarthritis or rheumatoid arthritis. J. Oral Maxillofac. Surg..

[111] Trieger N., Hoffman C.H., Rodriguez E. (1999). The effect of arthrocentesis of the temporomandibular joint in patients with rheumatoid arthritis. J. Oral Maxillofac. Surg..

[112] Antonarakis G.S., Courvoisier D.S., Hanquinet S., Dhouib A., Carlomagno R., Hofer M., Scolozzi P. (2018). Benefit of temporomandibular joint lavage with intra-articular steroids versus lavage alone in the management of temporomandibular joint involvement in juvenile idiopathic arthritis. J. Oral Maxillofac. Surg..

[113] Vallon D., Akerman S., Nilner M., Petersson A. (2002). Long-term follow-up of intra-articular injections into the temporomandibular joint in patients with rheumatoid arthritis. Swed. Dent. J..

[114] Ringold S., Thapa M., Shaw E.A., Wallace C.A. (2011). Heterotopic ossification of the temporomandibular joint in juvenile idiopathic arthritis. J. Rheumatol..

[115] Stoll M.L., Amin D., Powell K.K., Poholek C.H., Strait R.H., Aban I., Beukelman T., Young D.W., Cron R.Q., Waite P.D. (2018). Risk Factors for Intraarticular Heterotopic Bone Formation in the Temporomandibular Joint in Juvenile Idiopathic Arthritis. J. Rheumatol..

[116] O’Connor R.C., Fawthrop F., Salha R., Sidebottom A.J. (2017). Management of the temporomandibular joint in inflammatory arthritis: Involvement of surgical procedures. Eur. J. Rheumatol..

[117] Kopp S., Akerman S., Nilner M. (1991). Short-term effects of intra-articular sodium hyaluronate, glucocorticoid, and saline injections on rheumatoid arthritis of the temporomandibular joint. J. Craniomandib. Disord..

[118] Alstergren P., Larsson P.T., Kopp S. (2008). Successful treatment with multiple intra-articular injections of infliximab in a patient with psoriatic arthritis. Scand. J. Rheumatol..

[119] Stoll M.L., Morlandt A.B., Teerawattanapong S., Young D., Waite P.D., Cron R.Q. (2013). Safety and efficacy of intra-articular infliximab therapy for treatment-resistant temporomandibular joint arthritis in children: A retrospective study. Rheumatology.

[120] Stoll M.L., Vaid Y.N., Guleria S., Beukelman T., Waite P.D., Cron R.Q. (2015). Magnetic resonance imaging findings following intraarticular infliximab therapy for refractory temporomandibular joint arthritis among children with juvenile idiopathic arthritis. J. Rheumatol..

[121] Kristensen K.D., Stoustrup P., Küseler A., Pedersen T.K., Nyengaard J.R., Hauge E., Herlin T. (2009). Intra-articular vs. systemic administration of etanercept in antigen-induced arthritis in the temporomandibular point. Part I: Histological effects. Pediatr. Rheumatol. Online J..

[122] Sperry M.M., Yu Y.H., Kartha S., Ghimire P., Welch R.L., Winkelstein B.A., Granquist E.J. (2020). Intra-articular etanercept attenuates pain and hypoxia from TMJ loading in the rat. J. Orthop. Res..

[123] Bjørnland T., Larheim T.A. (1995). Synovectomy and diskectomy of the temporomandibular joint in patients with chronic arthritic disease compared with diskectomies in patients with internal derangement. A 3-year follow-up study. Eur. J. Oral Sci..

[124] Bjornland T., Larheim T.A., Haanaes H.R. (1992). Surgical treatment of temporomandibular joints in patients with chronic arthritic disease: Preoperative findings and one-year follow-up. Cranio.

[125] Leshem D., Tompson B., Britto J.A., Forrest C.R., Phillips J.H. (2006). Orthognathic surgery in juvenile rheumatoid arthritis patients. Plast. Reconstr. Surg..

[126] Pagnoni M., Amodeo G., Fadda M.T., Brauner E., Guarino G., Virciglio P., Iannetti G. (2013). Juvenile idiopathic/rheumatoid arthritis and orthognathic surgery without mandibular osteotomies in the remittent phase. J. Craniofac. Surg..

[127] Oye F., Bjørnland T., Støre G. (2003). Mandibular osteotomies in patients with juvenile rheumatoid arthritic disease. Scand. J. Rheumatol..

[128] Thaller S.R., Cavina C., Kawamoto H.K. (1990). Treatment of orthognathic problems related to scleroderma. Ann. Plast. Surg..

[129] Mercuri L.G., Handelman C.S. (2020). Idiopathic condylar resorption: What should we do?. Oral Maxillofac. Surg. Clin. N. Am..

[130] Nørholt S.E., Pedersen T.K., Herlin T. (2013). Functional changes following distraction osteogenesis treatment of asymmetric mandibular growth deviation in unilateral juvenile idiopathic arthritis: A prospective study with long-term follow-up. Int. J. Oral Maxillofac. Surg..

[131] Mackool R.L., Shetye P., Grayson B., McCarthy J.G. (2006). Distraction osteogenesis in a patient with juvenile arthritis. J. Craniofac. Surg..

[132] Ferguson J.W., Luyk N.H., Parr N.C. (1993). A potential role for costo-chondral grafting in adults with mandibular condylar destruction secondary to rheumatoid arthritis—A case report. J. Craniomaxillofac. Surg..

[133] MacIntosh R.B., Shivapuja P.K., Naqvi R. (2015). Scleroderma and the temporomandibular joint: Reconstruction in 2 variants. J. Oral Maxillofac. Surg..

[134] Felix V.B., Cabral D.R., de Almeida A.B., Soares E.D., de Moraes Fernandes K.J. (2017). Ankylosis of the temporomandibular joint and reconstruction with a costochondral graft in a patient with juvenile idiopathic arthritis. J. Craniofac. Surg..

[135] Keyser B.R., Banda A.K., Mercuri L.G., Warburton G., Sullivan S.M. (2020). Alloplastic total temporomandibular joint replacement in skeletally immature patients: A pilot survey. Int. J. Oral Maxillofac. Surg..

[136] Sidebottom A.J. (2008). UK TMJ replacement surgeons; British Association of Oral and Maxillofacial Surgeons. Guidelines for the replacement of temporomandibular joints in the United Kingdom. Br. J. Oral Maxillofac. Surg..

[137] Mehra P., Henry C.H., Giglou K.R. (2018). Temporomandibular joint reconstruction in patients with autoimmune/connective tissue disease. J. Oral Maxillofac. Surg..

[138] Mehra P., Wolford L.M., Baran S., Cassano D.S. (2009). Single-stage comprehensive surgical treatment of the rheumatoid arthritis temporomandibular joint patient. J. Oral Maxillofac. Surg..

[139] Hechler B.L., Matthews N.S. (2021). Role of alloplastic reconstruction of the temporomandibular joint in the juvenile idiopathic arthritis population. Br. J. Oral Maxillofac. Surg..

[140] Brown Z., Rushing D.C., Perez D.E. (2020). Alloplastic temporomandibular joint reconstruction for patients with juvenile idiopathic arthritis. J. Oral Maxillofac. Surg..

[141] Mishima K., Yamada T., Sugahara T. (2003). Evaluation of respiratory status and mandibular movement after total temporomandibular joint replacement in patients with rheumatoid arthritis. Int. J. Oral Maxillofac. Surg..

[142] Saeed N.R., McLeod N.M., Hensher R. (2001). Temporomandibular joint replacement in rheumatoid-induced disease. Br. J. Oral Maxillofac. Surg..

[143] O’Connor R.C., Saleem S., Sidebottom A.J. (2016). Prospective outcome analysis of total replacement of the temporomandibular joint with the TMJ Concepts system in patients with inflammatory arthritic diseases. Br. J. Oral Maxillofac. Surg..

[144] Felstead A.M., Revington P.J. (2011). Surgical management of temporomandibular joint ankylosis in ankylosing spondylitis. Int. J. Rheumatol..

[145] Manemi R.V., Fasanmade A., Revington P.J. (2009). Bilateral ankylosis of the jaw treated with total alloplastic replacement using the TMJ concepts system in a patient with ankylosing spondylitis. Br. J. Oral Maxillofac. Surg..

[146] Balon P., Vesnaver A., Kansky A., Kočar M., Prodnik L. (2019). Treatment of end stage temporomandibular joint disorder using a temporomandibular joint total prosthesis: The Slovenian experience. J. Craniomaxillofac. Surg..

[147] Lypka M., Shah K., Jones J. (2020). Prosthetic temporomandibular joint reconstruction in a cohort of adolescent females with juvenile idiopathic arthritis. Pediatr. Rheumatol. Online J..

[148] Goodman S.M., Springer B., Guyatt G., Abdel M.P., Dasa V., George M., Gewurz-Singer O., Giles J.T., Johnson B., Lee S. (2017). 2017 American College of Rheumatology/American Association of Hip and Knee Surgeons guideline for the perioperative management of antirheumatic medication in patients with rheumatic diseases undergoing elective total hip or total knee arthroplasty. J. Arthroplast..

